# Cardiotrophin-1 Deficiency Abrogates Atherosclerosis Progression

**DOI:** 10.1038/s41598-020-62596-6

**Published:** 2020-04-01

**Authors:** Kapka Miteva, Daniela Baptista, Fabrizio Montecucco, Mohamed Asrih, Fabienne Burger, Aline Roth, Rodrigo A. Fraga-Silva, Nikolaos Stergiopulos, François Mach, Karim J. Brandt

**Affiliations:** 1 Division of Cardiology, Foundation for Medical Research, Department of Medicine Specialized Medicine, Faculty of Medicine, University of Geneva, Av. de la Roseraie 64, CH-1211 Geneva 4, Switzerland; 2 Ospedale Policlinico San Martino Genoa – Italian Cardiovascular Network, 10 Largo Benzi, Genoa, 16132 Italy; 3 First Clinic of Internal Medicine, Department of Internal Medicine and Centre of Excellence for Biomedical Research (CEBR), University of Genoa, 6 viale Benedetto XV, Genoa, 16132 Italy; 4 Institute of Bioengineering, Ecole Polytechnique Fédérale de Lausanne, Lausanne, Switzerland

**Keywords:** Interleukins, Atherosclerosis

## Abstract

Cardiotrophin-1 (CT-1) is associated with cardiovascular (CV) diseases. We investigated the effect of CT-1 deficiency in the development and progression of atherosclerosis in double knockout Apoe^−/−^ct-1^−/−^ mice. Apoe^−/−^ C57Bl/6 or Apoe^−/−^ct-1^−/−^ C57Bl/6 mice were fed a normal chow diet (NCD) or a high-cholesterol diet (HCD). After sacrifice, serum triglycerides, total cholesterol, low-density lipoprotein cholesterol (LDL-C), free fatty acids and systemic paracrine factors were measured. Intraplaque lipid and collagen content were quantified in the aortic sections. Immune cell populations in spleen, lymph nodes and aorta were analysis by flow cytometry. Apoe^−/−^ct-1^−/−^ mice in accelerated atherosclerosis exhibited a reduction of total cholesterol, LDL-C, atherosclerotic plaques size in the aortic root and in the abdominal aorta and improved plaque stability in comparison to Apoe^−/−^ mice. CT-1 deficiency in Apoe^−/−^ mice on (HCD) promoted atheroprotective immune cell responses, as demonstrated by a rise in plasma anti-inflammatory immune cell populations (regulatory T cells, Tregs; regulatory B cells, Bregs and B1a cells) and atheroprotective IgM antibodies. CT-1 deficiency in advanced atherosclerosis mediated regulation of paracrine factors, such as interleukin (IL)-3, IL-6, IL-9, IL-15, IL-27, CXCL5, MCP-3, MIP-1α and MIP-1β. In a model of advanced atherosclerosis, CT-1 deficiency induced anti-inflammatory and atheroprotective effects which resulted in abrogation of atheroprogression.

## Introduction

Cardiovascular (CV) diseases are the leading cause of death and morbidity in developed countries^1^. The underlying cause of the most serious CV events is atherosclerosis, which is defined as a chromic inflammatory disease characterized by the build-up of subendothelial cholesterol deposits and the formation of leukocyte-rich plaques in the intimal layer of arteries. The fibrous cap is an atheroprotective layer of vascular smooth muscle cells (VSMCs) that covers the atherosclerotic plaque^2^ and induces acute thrombo-occlusive events, such as myocardial infarction and stroke^3^. Immune cells and inflammation play a key role in promoting the disruption of the fibrous cap^2^. Full comprehension of the mechanisms of atherosclerosis is linked to revealing the role of the paracrine mediators released by the heterogenous cell populations involved in the development, progression, and complications of atherosclerosis.

Cardiotrophin-1 (CT-1) is a member of the interleukin (IL)-6 family of cytokines, and was initially cloned based on its ability to induce hypertrophy in neonatal cardiomyocytes^4^. CT-1 is highly expressed in the cells of the cardiovascular system – cardiomyocytes, cardiac fibroblasts, vascular endothelial cells, vascular smooth muscle cells (VSMCs), macrophages^5–8^, as well as in other organs^9,10^. Factors like mechanical stretching, hypoxia, angiotensin II, aldosterone, growth factors, insulin, glucose, reactive oxygen species^11–16^, hypertension^17^ and pressure overload^18^ trigger CT-1 expression. CT-1 binds to glycoprotein 130 (gp130) and leukemia inhibitory factor receptor (LIFR)^4^ and induces cardiac protection via inhibition of apoptosis, suggesting a protective role of CT-1 in response to acute hypoxia *in vivo*^19^ which is in line with other studies showing the anti-apoptotic effect of CT-1 treatment on embryonic, neonatal, and adult cardiomyocytes^6,20,21^. On the other hand CT-1 has been shown promotes cardiac hypertrophy^22^, atherosclerosis, arterial stiffness^23^ and vascular inflammation^5^. Elevated CT-1 levels are detected in the serum of patients with heart failure^24^ and coronary artery disease^25^. CT-1 promotes atherogenesis via activation of endothelial cells^8^, stimulation of monocyte endothelial cells adhesion and monocyte migration^8^. CT-1 triggers inflammatory and proatherogenic molecule expression, such as IL-6^26^, monocyte chemoattractant protein-1, intercellular adhesion molecule-1, matrix metalloproteinase-1 (MMP-1)^8,27,28^, IL-1β, and tumor necrosis factor-α in monocytes^29^. In addition, CT-1 promotes the formation of foam cells^5^ and stimulates migration, proliferation^5^, apoptosis and senescence in VSMCs^30^. Moreover, infusion of CT-1 into Apoe^−/−^ mice for four weeks directly escalated the development of aortic atherosclerotic lesions with increased monocyte/macrophage infiltration and VSMCs cell proliferation^5^.

Although it has been shown that CT-1 accelerates atherosclerosis, its role in the pathogenesis of atherosclerosis is still not established. In the present study, we investigated the effect of CT-1 inhibition in atherosclerosis in double knockout Apoe^−/−^ct-1^−/−^ mice. In particular, we evaluated the impact of CT-1 deficiency on atherosclerotic lesion size, plaque stability, cholesterol levels and inflammation.

## Results

### CT-1 Modulates cholesterols levels in Apoe^−/−^ mice on high cholesterol diet

**Figure 1:**
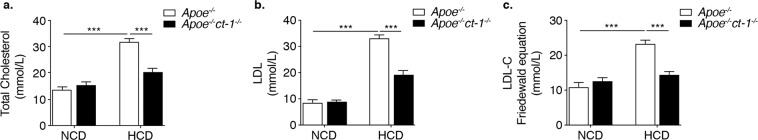
Apoe^−/−^ct-1^−/−^ Mice on High Cholesterol Diet Exhibit Lower Cholesterol Levels Bar graphs represent the mean ± SEM of (**a**) total cholesterol; (**b**) LDL; and (**c**) LDL-C, calculated by Friedewald equation and expressed as mmol/L in Apoe^−/−^ and Apoe^−/−^ct-1^−/−^ mice on NCD or HCD, as indicated, with n  =  6–8/group and ***p  <  0.001.

Apoe^−/−^ mice or Apoe^−/−^ct-1^−/−^ mice were fed NCD for 16 weeks or HCD for 11 weeks and the levels of cholesterol were quantified. HCD significantly increased the levels of total cholesterol, LDL and LDL-C in Apoe^−/−^ mice (Fig. 1). However, in comparison to Apoe^−/−^ mice on HCD, CT-1-deficient Apoe^−/−^ mice on HCD exhibited a 1.6-fold and 1.7-fold reduction of total cholesterol and LDL, respectively (Fig. 1a,b). LDL-C was calculated by Friedewald formula, which estimates LDL levels by taking into account the levels of total cholesterol, triglycerides and LDL, and was diminished 1.6-fold in Apoe^−/−^ct-1^−/−^ mice versus Apoe^−/−^ mice on HCD (Fig. 1c). LDL levels are a causal factor in atherosclerosis, therefore, the effect of CT-1 deficiency on LDL levels in accelerated atherosclerosis is an important prerequisite for the improvement of cardiovascular outcomes.

### CT-1 deficiency reduces atherosclerosis lesion size and modulates parameters of plaque stability in Apoe^−/−^ mice on high cholesterol diet

**Figure 2:**
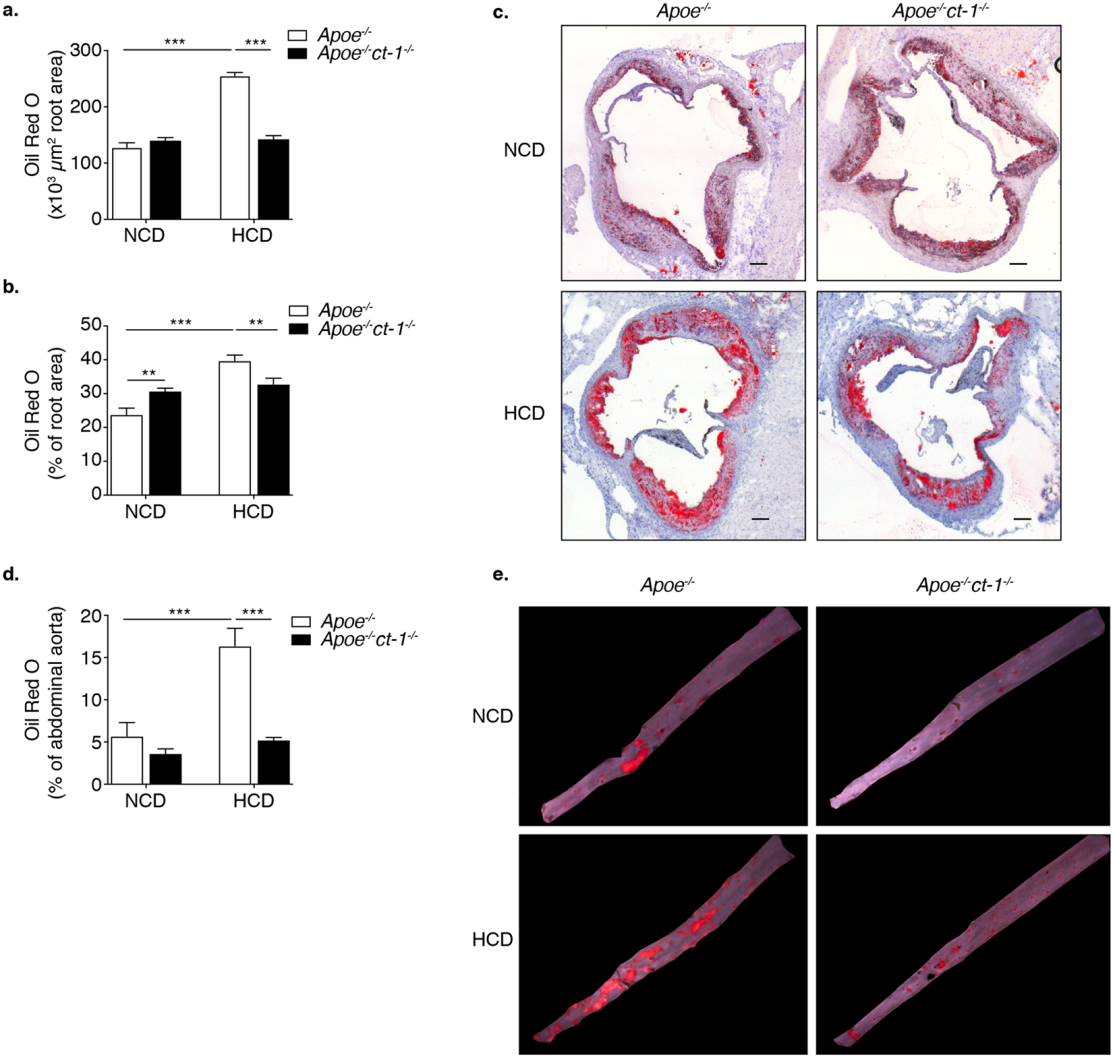
CT-1 Deficiency in Apoe^−/−^ Mice Promotes Reduction in Atherosclerotic Lesion Size in Accelerated Atherosclerosis Bar graphs represent the mean ± SEM of Oil Red O quantification of (**a)** atherosclerotic lesion size, expressed as μm^2^ in aortic roots; and (**b**) atherosclerotic lesion size, expressed as % of total aortic root area in Apoe^−/−^ or Apoe^−/−^ct-1^−/−^ mice on NCD or HCD, as indicated, with n  =  6–8/group and **p  <  0.01, ***p  <  0.001. (**c**) Representative pictures of Oil Red O stained atherosclerotic lesions in the roots of Apoe^−/−^ or Apoe^−/−^ct-1^−/−^ mice on NCD (upper panel) or HCD (lower panel). (**d**) Bar graphs represent the mean ± SEM of Oil Red O quantification of atherosclerotic lesion size, expressed as % of abdominal aorta area in Apoe^−/−^ or Apoe^−/−^ct-1^−/−^ mice on NCD or HCD, as indicated, with n  =  6–8/group and ***p  <  0.001. (**e**) Representative pictures of Oil Red O stained atherosclerotic lesions of the abdominal aorta of Apoe^−/−^ or Apoe^−/−^ct-1^−/−^ mice on NCD (upper panel) or HCD (lower panel). The scale bar is 200 μm.

**Figure 3:**
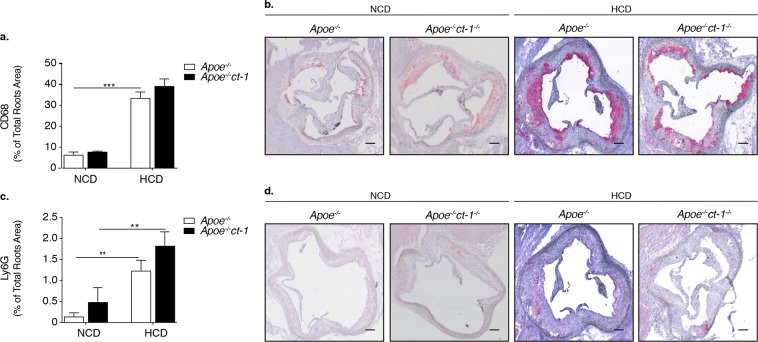
CD68 and Ly6G Expression in Aortic Roots is not Affected by CT-1 deficiency. (**a**) Bar graphs represent the mean ± SEM of CD68 expressed as % of total aortic root area in Apoe^−/−^ or Apoe^−/−^ct-1^−/−^ mice on NCD or HCD, and (**b**) representative pictures of CD68 staining in atherosclerotic roots in Apoe^−/−^ or Apoe^−/−^ct-1^−/−^ mice on NCD or HCD, as indicated, with n  =  6–8/group and ***p  <  0.001. (**c**) Bar graphs represent the mean ± SEM of Ly6G expressed as % of total aortic root area in Apoe^−/−^ or Apoe^−/−^ct-1^−/−^ mice on NCD or HCD, and (**d**) representative pictures of Ly6G staining in atherosclerotic roots in Apoe^−/−^ or Apoe^−/−^ct-1^−/−^ mice on NCD or HCD, as indicated, with n  =  6–8/group and **p  <  0.01. The scale bar is 200 μm.

**Figure 4:**
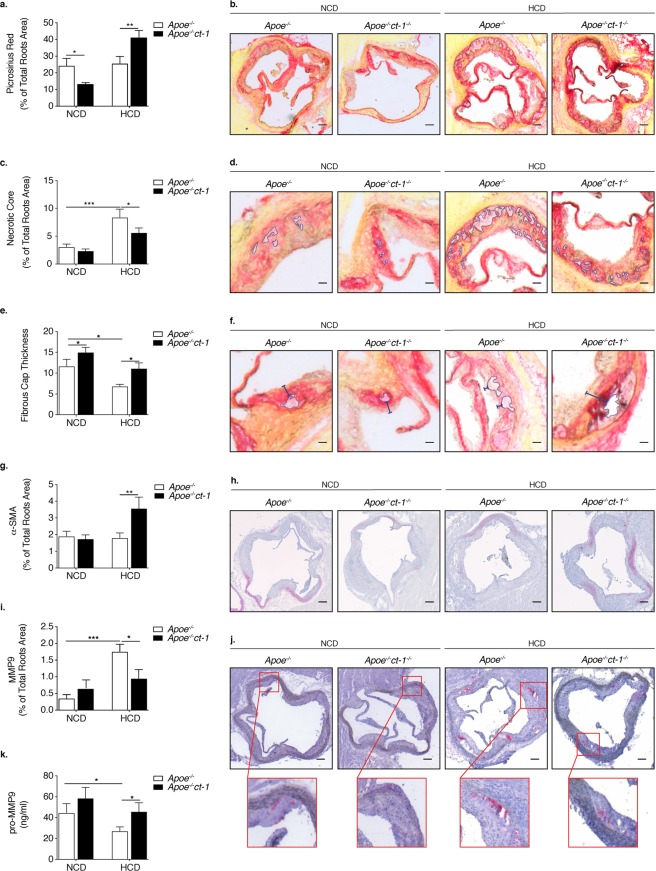
Lack of CT-1 in Accelerated Atherosclerosis Improves Parameters Associated with Plaque Stability. Bar graphs represent the mean ± SEM of (**a**) Picrosirius Red quantification of total collagen, expressed as % of aortic root area; and (**b**) representative pictures of Picrosirius Red staining of total collagen in atherosclerotic roots in Apoe^−/−^ or Apoe^−/−^ct-1^−/−^ mice on NCD or HCD, as indicated, with n = 6–8/group and *p  <  0.05 and **p  <  0.01, expressed as % of aortic root area. The scale bar is 200 μm. (**c**) Bar graphs represent the mean ± SEM of necrotic core expressed as % of total aortic root area in Apoe^−/−^ or Apoe^−/−^ct-1^−/−^ mice on NCD or HCD, as indicated, and (**d**) representative pictures of necrotic core quantification in atherosclerotic roots in Apoe^−/−^ or Apoe^−/−^ct-1^−/−^ mice on NCD or HCD, as indicated, with n  =  6–8/group and *p  <  0.05, and ***p  <  0.001 expressed as % of aortic root area. The scale bar is 200 μm. (**e**) Bar graphs represent the mean ± SEM of fibrous cap thickness in Apoe^−/−^ or Apoe^−/−^ct-1^−/−^ mice on NCD or HCD, as indicated, and (**f**) representative pictures of necrotic core thickness quantification in atherosclerotic roots in Apoe^−/−^ or Apoe^−/−^ct-1^−/−^ mice on NCD or HCD, as indicated, with n ^=^ 6–8/group and *p  <  0.05, and ***p  <  0.001 expressed as % of aortic root area. The scale bar is 200 μm. (**g**) Bar graphs represent the mean ± SEM of α-SMA expressed as % of total aortic root area in Apoe^−/−^ or Apoe^−/−^ct-1^−/−^ mice on NCD or HCD, as indicated, and (**h**) representative pictures of α-SMA staining in atherosclerotic roots in Apoe^−/−^ or Apoe^−/−^ct-1^−/−^ mice on NCD or HCD, as indicated, with n  =  6–8/group and ^**^p  <  0.01 expressed as % of aortic root area. (**i**) Bar graphs represent the mean ± SEM of MMP9 expressed as % of aortic root area in Apoe^−/−^ or Apoe^−/−^ct-1^−/−^ mice on NCD or HCD, as indicated, and (**j**) representative pictures of MMP9 in atherosclerotic roots in Apoe^−/−^ or Apoe^−/−^ct-1^−/−^ mice on NCD or HCD, as indicated, with n  =  6–8/group and *p  <  0.05, and ***p  <  0.001 expressed as % of aortic root area. (**k**) Bar graphs represent the mean ± SEM of pro-MMP9 quantification in the serum of Apoe^−/−^ or Apoe^−/−^ct-1^−/−^ mice on NCD or HCD, as indicated, with n  =  6–8/group and *p  <  0.05. The scale bar is 200 μm.

To determine the pathological function of CT-1 in atherogenesis, we examined whether CT-1 deletion alters atherosclerotic plaque size and stability. Apoe^−/−^ mice and Apoe^−/−^ct-1^−/−^ mice were fed NCD for 16 weeks or HCD for 11 weeks. Atherosclerotic lesion size in the aortic roots was significantly increased in Apoe^−/−^ mice on HCD versus Apoe^−/−^ mice on NCD (Fig. 2a,b), and as also evident in the representative picture of Oil Red O staining (Fig. 2c). In contrast, Apoe^−/−^ct-1^−/−^ mice on HCD exhibited a 1.8-fold reduction in lesion size area in the aortic root (Fig. 2a) versus Apoe^−/−^ mice on HCD. Atherosclerotic lesion size, expressed as a percentage in the aortic roots and abdominal part of the aorta, was significantly increased in Apoe^−/−^ mice on HCD versus Apoe^−/−^ mice on NCD (Fig. 2b,d). During spontaneous atherosclerosis, Apoe^−/−^ct-1^−/−^ mice showed a 1.2-fold increase in lesion size compared to Apoe^−/−^ mice on NCD, expressed as the percentage of total lesion area in the aortic root (Fig. 2b). However, in accelerated atherosclerosis (HCD), lesion size reduction in Apoe^−/−^ct-1^−/−^ versus Apoe^−/−^ mice was 1.2-fold in the aortic root (Fig. 2b) and 3.2-fold in the abdominal aorta (Fig. 2d), as illustrated in the representative images of Oil Red O staining (Fig. 2c,e). HCD induced significant increase in the percentage of lesion CD68 macrophages (Fig. 3a,b) and neutrophils Ly6G cells (Fig. 3c,d) in the aortic roots of Apoe^−/−^ mice, while CT-1 deficiency had not effect on macrophages or neutrophils accumulation in the aortic roots under NCD or HCD. Collagen plays a key role in determining plaque stability and protects plaque against rupture^31^. Picrosirius red staining of the atherosclerotic lesions in the aortic roots revealed a significant reduction of collagen accumulation in Apoe^−/−^ct-1^−/−^ versus Apoe^−/−^ mice on NCD (Fig. 4a,b). However, in accelerated atherosclerosis the collagen content was increased by 1.6-fold in Apoe^−/−^ct-1^−/−^ versus Apoe^−/−^ mice on HCD (Fig. 4a,b). In parallel, CT-1 deficiency in Apoe^−/−^ on HCD resulted in a significant reduction in the necrotic core area (Fig. 4c,d) and an increase in the fibrous core thickness in Apoe^−/−^ct-1^−/−^ mice on both NCD and HCD (Fig. 4e,f) versus Apoe^−/−^ mice. Importantly, the increase in the collagen content in Apoe^−/−^ct-1^−/−^ mice on HCD was associated with a prominent increase of 2-fold α-SMA expression in the atherosclerotic plaques of Apoe^−/−^ct-1^−/−^ mice (Fig. 4g,h) versus Apoe^−/−^ mice on HCD. In addition, Apoe^−/−^ct-1^−/−^ exhibited on HCD a 1.9-fold reduction in MMP9 expression in the atherosclerotic roots versus Apoe^−/−^ mice (Fig. 4i,j). Interestingly, the levels of pro-MMP-9 in the serum of Apoe^−/−^ mice on HCD were reduced versus Apoe^−/−^ mice on NCD, while Apoe^−/−^ct-1^−/−^ mice on HCD had significantly higher pro-MMP9 levels (Fig. 4k), indicating a possible reduction in the proteolysis of the pro-peptide to active MMP9 in the serum of Apoe^−/−^ct-1^−/−^ mice on HCD. The present findings indicate that CT-1 deficiency in Apoe^−/−^ mice in accelerated atherosclerosis not only results in reduced atherosclerotic lesion size, but it also modulates key aspects of the plaque structure stability with major clinical consequences.

### CT-1 Deficiency affects immune cell response

**Figure 5:**
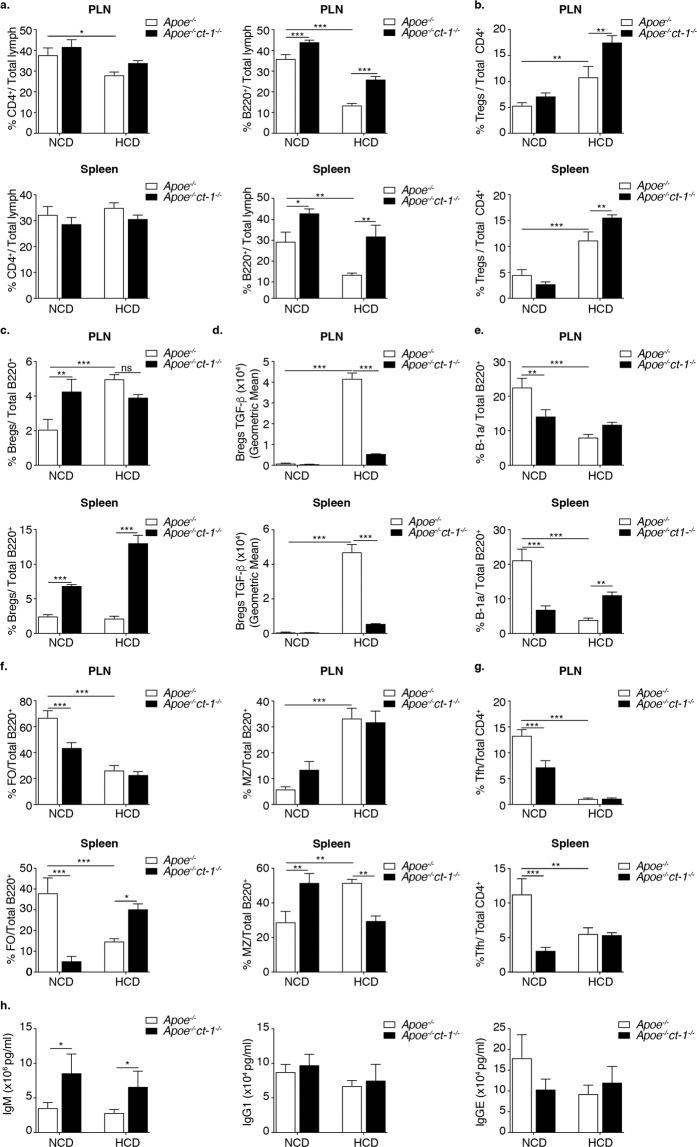
CT-1 Deficiency Promotes Atheroprotective Immune Cell Response. Bar graphs represent the mean ± SEM of flow cytometry analysis of (**a**) CD4^+^ and B220^+^ cell percentages; **(b**) Tregs (CD4+FoxP3+) percentage; **(c**) Bregs percentage; (**d**) Bregs TGF-β geometric mean; **(e**) B1a cell percentage; **(f**) FOB and MZB cell percentages; (**g**) T_FH_ percentage in PLN upper panel and SP lower panel; and (**h**) IgM, IgG1 and IgGE quantification in the serum of Apoe^−/−^ and Apoe^−/−^ct-1^−/−^ mice on NCD or HCD, as indicated, with n  =  6–8/group and *p  <  0.05, **p  <  0.01 and ***p  <  0.001.

Apoe^−/−^ and Apoe^−/−^ct-1^−/−^ mice were fed either NCD or HCD and the immunomodulatory effects of CT-1 abrogation were assessed. CD4 cells decreased in the peripheral lymph nodes (PLN) of Apoe^−/−^ mice on HCD versus NCD (Fig. 5a). CT-1 deficiency did not affect CD4 cell counts in the PLN or spleen (SP) (Fig. 5a). B220^+^ cells were reduced in the PLN and SP of Apoe^−/−^ mice on HCD versus NCD (Fig. 5a). Apoe^−/−^ct-1^−/−^ mice on HCD showed an increase in B220^+^ cells in the PLN and SP versus Apoe^−/−^ on HCD (Fig. 5a). Regulatory T cells (Tregs) in the PLN and SP were significantly elevated in Apoe^−/−^ mice on HCD versus NCD (Fig. 5b). Moreover, Apoe^−/−^ct-1^−/−^ mice on HCD showed an increase in Tregs both in the PLN (1.6-fold) and SP (1.4-fold) (Fig. 5b). HCD fed Apoe^−/−^ mice exhibited significant increase in Th1, Th2 and Th17 cells in the PLN (Fig. S2a–c) and Th1 and Th2 in the spleen (Fig. S2d,e) versus NCD Apoe^−/−^ mice, while CT-1 deficiency in Apoe^−/−^ mice reduced the percentage of Th1, Th2 and Th17 cells under HCD in PLN and SP and Th17 cells also under NCD in PLN and spleen (Fig. S2). Regulatory B cells (Bregs) in the PLN increased in Apoe^−/−^ct-1^−/−^ mice on NCD and in Apoe^−/−^ on HCD versus Apoe^−/−^ mice on NCD (Fig. 5c). In addition, Apoe^−/−^ct-1^−/−^ mice on HCD exhibited a 6.8-fold increase in Bregs in the SP in comparison to Apoe^−/−^ mice on HCD (Fig. 5c). Interestingly, HCD in Apoe^−/−^ mice induced a pronounced increase in Bregs-produced TGF-β in the PLN and SP (Fig. 5d). However, CT-1 deficiency in Apoe^−/−^ mice on HCD induced a significant reduction of Bregs-produced TGF-β in the PLN (7.8-fold) and SP (8.7-fold) (Fig. 5d). B1a cells decreased in the PLN and SP of Apoe^−/−^ct-1^−/−^ mice on NCD and Apoe^−/−^ mice on HCD versus Apoe^−/−^ mice on NCD (Fig. 5e). Importantly, in accelerated atherosclerosis Apoe^−/−^ct-1^−/−^ mice showed a 2.8-fold increase in atheroprotective B1a cells in the SP versus Apoe^−/−^ mice on HCD (Fig. 5e). Follicular B (FOB) in the PLN and SP diminished in Apoe^−/−^ct-1^−/−^ mice on NCD and in Apoe^−/−^ mice on HCD versus Apoe^−/−^ mice on NCD (Fig. 5f), while in Apoe^−/−^ct-1^−/−^ mice on HCD, FOB cells in the SP were elevated compared to Apoe^−/−^ mice on HCD. In addition, Marginal zone B (MZB) cells in the PLN and SP were elevated in Apoe^−/−^ mice on HCD versus NCD (Fig. 5f). Furthermore, Apoe^−/−^ct-1^−/−^ mice on NCD exhibited an increase in MZB cells in the SP in comparison to Apoe^−/−^ mice on NCD, but MZB cells in the SP were significantly diminished in Apoe^−/−^ct-1^−/−^ mice on HCD versus Apoe^−/−^ mice on HCD (Fig. 5f). The follicular T (T_FH_) cells were prominently reduced in the PLN and SP of Apoe^−/−^ct-1^−/−^ mice on NCD and Apoe^−/−^ mice on HCD versus Apoe^−/−^ mice on NCD, while the percentage of T_FH_ cells in Apoe^−/−^ct-1^−/−^ mice on HCD was comparable to that observed in Apoe^−/−^ mice on HCD (Fig. 5g). Importantly, Apoe^−/−^ct-1^−/−^ mice on HCD showed a 2.3-fold increase in IgM concentration versus Apoe^−/−^ mice on HCD, while IgG1 and IgE levels remained unchanged in all conditions (Fig. 5h). It was thus shown that CT-1 deficiency in atherosclerosis has a pronounced effect on numerous immune cells populations. Interestingly, Apoe^−/−^ct-1^−/−^ mice on NCD and on HCD exhibited significant decrease in the oxLDL-IgM levels systemically in comparison to Apoe^−/−^ mice (Fig. S3).

### CT-1 deficiency in Apoe^−/−^ mice affects systemic inflammation

**Figure 6:**
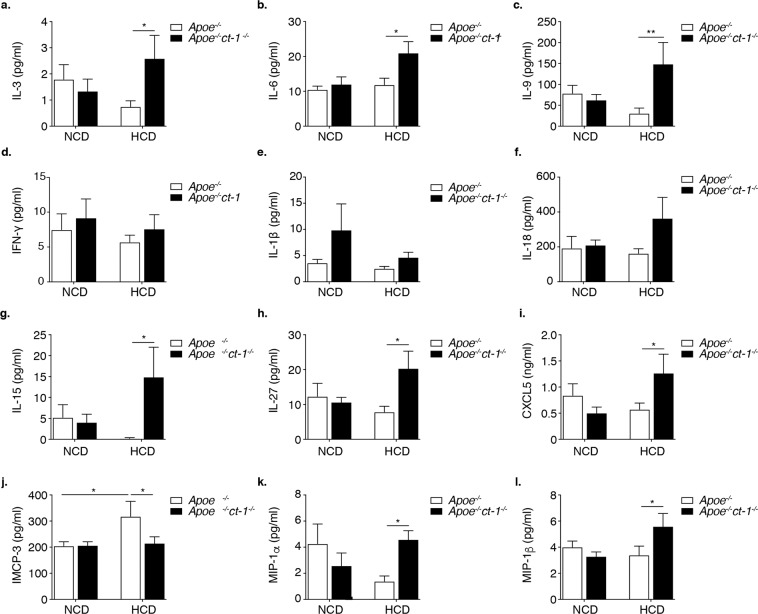
CT-1 Deficiency in Apoe^−/−^ mice in Accelerated Atherosclerosis Affects Systemic Inflammation. Bar graphs represent the mean ± SEM of multiplex immunoassay quantification of (**a**) lL-3; (**b**) IL-6; (**c**) IL-9; (**d**) IL-15; (**e**) IFN-γ; (**f**) IL-1β; (**g**) IL-18; (**h**) IL-27; (**i**) CXCL5; (**j**) MCP-3; (**k**) MIP-1α; and (**l**) MIP-1β, expressed as pg/ml in the serum of Apoe^−/−^ or Apoe^−/−^ct-1^−/−^ mice on NCD or HCD, as indicated, with n  =  6–8/group and *p  <  0.05 and **p  <  0.01.

Taking into account the importance of chronic inflammation in atherosclerosis, we investigated the impact of CT-1 deficiency on systemic immune mediators. The serum levels of cytokines and chemokines of Apoe^−/−^ and Apoe^−/−^ct-1^−/−^ mice fed NCD for 16 weeks or HCD for 11 weeks were quantified. Apoe^−/−^ct-1^−/−^ mice on HCD had a 5.1-fold increase in the systemic level of IL-3 (Fig. 6a) and a 2-fold increase in IL-6 in comparison to Apoe^−/−^ mice on HCD (Fig. 6b). Furthermore, ct-1^−/−^ deficient Apoe^−/−^ mice on HCD had a 4.8-fold higher concentration of IL-9 (Fig. 6c) in the serum versus Apoe^−/−^ mice. IFN-γ, IL-1β and IL-18 levels in the circulation did not differ significantly between the groups (Fig. 6d–f). However, ct-1^−/−^ deficient Apoe^−/−^ mice on HCD had a 4.8-fold higher concentration of IL-9 (Fig. 6c) in the serum versus Apoe^−/−^ mice. The abrogation of CT-1 in Apoe^−/−^ mice on HCD also affected the systemic levels of IL-15 and IL-27, as indicated by a 1.6-fold and 2.6-fold increase in the concentration of IL-15 and IL-27, respectively versus Apoe^−/−^ mice on HCD (Fig. 6d,e). Furthermore, CXCL5 systemic concentration was remarkably elevated in Apoe^−/−^ct-1^−/−^ mice versus Apoe^−/−^ mice on HCD in accelerated atherosclerosis (Fig. 6f). Systemic MCP-3 increased in Apoe^−/−^ mice on HCD vs NCD, while it decreased by 1.5-fold in Apoe^−/−^ct-1^−/−^ versus Apoe^−/−^ mice on HCD (Fig. 6g). We also observed 3.3- and 1.6-fold increases in the concentrations of MI1α and MI1β, respectively in Apoe^−/−^ct-1^−/−^ versus Apoe^−/−^ mice in accelerated atherosclerosis (Fig. 6h,i). These results suggest that CT-1 neutralization induces global systemic immunomodulatory effects.

### Tissue and cell specific expression of CT-1

**Figure 7:**
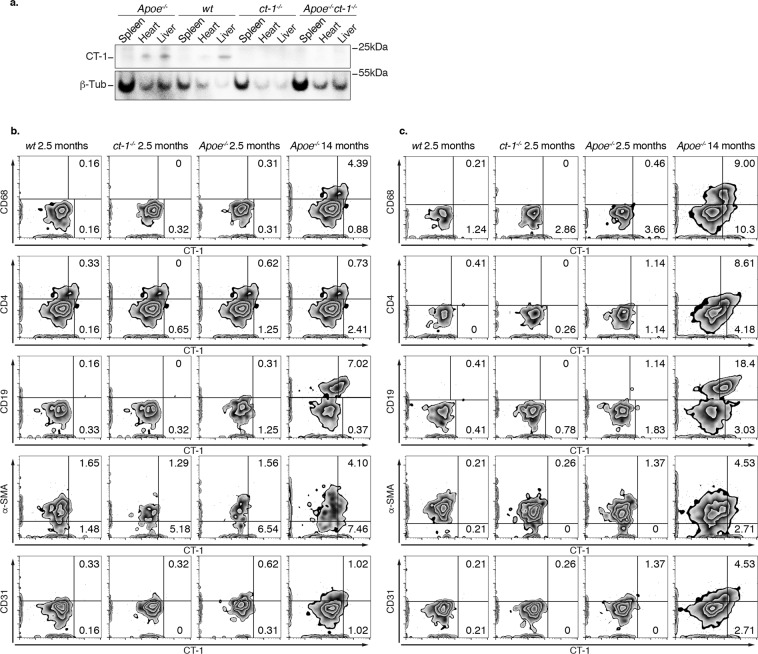
Tissue- and Cell-specific Expression of CT-1. Representative images of CT-1 expression analyzed (**a**) by Western blot in the SP, heart and liver of Apoe^−/−^, WT, ct-1^−/−^, and Apoe^−/−^ct-1^−/−^ mice; (**b**) by flow cytometry of CT-1 intracellularly; and (**c**) by surface staining in macrophages, CD4 T cells, CD19 cells, and α-SMA and CD31 positive cells in aorta cell suspensions of WT, ct-1^−/−^ and Apoe^−/−^ mice of 2.5 months and Apoe^−/−^ mice of 14 months on NCD.

To reveal the source of CT-1, we checked CT-1 expression in the SP, heart and liver of Apoe^−/−^, WT, ct-1^−/−^ and Apoe^−/−^ct-1^−/−^ mice. Apoe^−/−^ mice developed atherosclerotic plaques at the age of 14 months. As demonstrated in Fig. 7a, CT-1 expression was detected in the heart of Apoe^−/−^ mice and liver of Apoe^−/−^ and WT mice. Importantly ct-1^−/−^ or Apoe^−/−^ct-1^−/−^ mice did not express CT-1 in any of the investigated tissues. Cellular sources of CT-1 in the aorta of WT, ct-1^−/−^ and Apoe^−/−^ct-1^−/−^ and Apoe^−/−^ mice were investigated by flow cytometry analysis (Fig. 7b,c). CT-1 intracellular expression was detected in aorta macrophages, CD4 T cells, B-cells, VSMCs and endothelial cells of Apoe^−/−^ mice (Fig. 7b). Interestingly, CT-1 was highly expressed on the surface of aorta macrophages, CD4 T cells, B-cells, VSMCs and endothelial cells of Apoe^−/−^ mice developing atherosclerotic plaques at the age of 14 months (Fig. 7c).

## Discussion

The present study provides compelling evidence of the critical function of CT-1 in the pathogenesis of atherosclerosis. In accelerated atherosclerosis, CT-1 deficiency appears to induce a multitude of beneficial effects. Significantly, CT-1 abrogation not only resulted in lower cholesterol levels and smaller atherosclerotic plaques, but it also promoted systemic immunomodulatory effects, including 1) induction of atheroprotective immune cell populations – Tregs, Bregs and B1a cells; 2) regulation of systemic inflammatory factors; and 3) increased levels of circulating IgM, the concentration of which is known to be inversely correlated with atherosclerosis^32^. Moreover, Apoe^−/−^ct-1^−/−^ mice exhibited a number of changes in the fibrous cap formation known to be beneficial for plaque stability in atherosclerosis, in particular higher collagen accumulation, preserved α-SMA expression, and smaller necrotic cores, thicker fibrous caps and lower MMP9 expression in the aortic roots. Taken together, these observations have important implications. First, CT-1 undoubtedly emerges as a key factor in the pathogenesis of atherosclerosis; and second, CT-1 inhibition results in abrogation of atherosclerotic disease progression.

One of the most intriguing findings of the present study is the effect of CT-1 deficiency on the cholesterol levels and atherosclerotic plaque size in Apoe^−/−^ mice in accelerated atherosclerosis. Plasma total cholesterol and LDL levels are highly correlated with the extent of coronary atherosclerosis^33^, while hypercholesterolemia also enhances local and systemic proinflammatory responses^34^. Therefore, the reduction of LDL observed in Apoe^−/−^ct-1^−/−^ mice on HCD has two very significant consequences: on the one hand, it directly results in diminished LDL retention in the subendothelial space, leading to less atherosclerotic plaque formation; on the other hand, it indirectly leads to reduced inflammation. As a result, Apoe^−/−^ct-1^−/−^ mice on HCD exhibited atherosclerotic plaque sizes comparable to Apoe^−/−^ mice on NCD, however no difference in macrophages nor neutrophils accumulation in the roots was observed. Particularly impressive in accelerated atherosclerosis was the reduction in the size of the atherosclerotic lesions in the abdominal aorta of Apoe^−/−^ct-1^−/−^ mice. However, Apoe^−/−^ct-1^−/−^ mice develop bigger atherosclerotic lesions expressed as a percentage of roots area during spontaneous atherosclerosis, which could be probably associated with the anti-apoptotic effect mediated by CT-1^6,20,21^ and in response to acute hypoxia *in vivo*^19^. During early atherosclerosis, the reduction of apoptotic cells accumulation has been shown to limit local inflammation and lesion growth by preventing secondary cellular necrosis^35^, the increased lesion size in CT-1 deficient Apoe^−/−^ mice during spontaneous atherosclerosis could be linked to the abrogation of the anti-apoptotic effect of CT-1. However, our findings highlight, that deficiency of CT-1 becomes particularly beneficial in accelerated atherosclerosis where plaque complexity increases, and inflammatory and immune processes start to play an important role in plaque progression.

The majority of coronary thrombosis events are caused by plaque rupture^36^. The structure, composition, and turnover of the extracellular matrix (ECM) are crucial for atherosclerotic plaque stability. Picrosirius red staining of atherosclerotic lesions in the aortic sinus revealed a significant increase in collagen content in Apoe^−/−^ct-1^−/−^ mice with accelerated atherosclerosis, in parallel with a prominent reduction of MMP9 expression and necrotic core and increase in fibrous cap thickness and α-SMA expression in the atherosclerotic roots. A well-established dynamic equilibrium of ECM production and degradation is an essential prerequisite for determining plaque stability. In this regard, it is well known that collagen accumulation prevents plaque rupture^31^, while matrix-degrading proteases like MMP-9 degrade components of the extracellular matrix. MMP-9 is a protein expressed and secreted in an inactive form named pro-MMP-9, which is then activated by proteolysis of the propeptide domain^37^. MMP-9 is abundantly expressed in atherosclerotic plaques with a vulnerable histological appearance^38^. Interestingly, the levels of pro-MMP-9 in the serum of Apoe^−/−^ mice on HCD were tendentially reduced, while Apoe^−/−^ct-1^−/−^ mice on HCD showed upregulated pro-MMP9 levels, indicating a reduction in proteolysis of the propeptide to active MMP9 in the serum of Apoe^−/−^ct-1^−/−^ mice in accelerated atherosclerosis. CT-1 deficiency of Apoe^−/−^ mice in accelerated atherosclerosis not only diminished atherosclerotic lesion size, but it also improves the plaque structure stability parameters known to have a critical clinical importance due to atherosclerotic plaque ruptures.

Atherosclerosis is characterized by a chronic, low-grade inflammatory response associated with infiltration of immune cells into the atherosclerotic plaque^39^. Moreover, LDL acts as a self-antigen that drives an autoimmune response against self-proteins in the atherosclerotic plaque^40^. Importantly, CT-1 abrogation promoted Tregs cells, shown to mediate protective effects in atherosclerosis^41^ through the release of anti‐inflammatory cytokines and suppression of autoreactive effector T cells.

Moreover, CT-1 deficiency in Apoe^−/−^ mice has a profound effect on Th1, Th2 and Th17 cells under HCD and Th17 cells also under NCD. While Th1 cells are known to be highly inflammatory and to promote and accelerate lesion development^42^, the role of Th2 and Th17 cells is less clear, however CT-1 deficiency seems to greatly reduce the percentage of Th1, Th2 and Th17 systemically which could result in regulation in the systemic inflammation. In parallel, Bregs is known to exert vascular protective functions via the release of soluble factors, such as IL-10, TGF-β and IL-35^43^, and levels were increased in the SP of Apoe^−/−^ct-1^−/−^ mice on HCD. Conversely, it is known that increased levels of TGFβ1 correlate with accelerated atherosclerosis^44^ and induction of vascular calcification^45^. Importantly, Bregs producing TGF-β in the PLN and SP of Apoe^−/−^ct-1^−/−^ mice on HCD were reduced. This important observation indicates that the induction of atheroprotective Bregs does not lead to increased atherosclerosis, promoting TGF-β production. In accelerated atherosclerosis, Apoe^−/−^ct-1^−/−^ mice exhibited an expansion of the atheroprotective B1a cells in the SP, as well as elevated IgM^46,47^. B1a cells are known to secrete natural IgM, which is deposited in atherosclerotic lesions where it mediates necrotic core reduction^47^. However, it has been shown that the level of circulating oxLDL IgM autoAbs is lower in CAD patients than in no CAD patients, supporting the hypothesis that the oxLDL IgM autoAbs might be inversely associated with the presence of atherosclerosis^48^, while anti-oxLDL, anti-oxLDL-β2GPI IgM and anti-oxLDL-β2GPI IgG may increase the risk for cardiovascular diseases in systemic lupus erythematosus patients^49^. CT-1 deficiency promoted a significant reduction in the levels of IgM to oxLDL in Apoe^−/−^ mice, which is in line with the discussed below findings, that oxLDL IgM autoAbs might be inversely associated with the extent of atherosclerosis. However, the role of circulating oxLDL IgM remains controversial, since it has also been demonstrated that IgM to oxLDL could play a protective role too, however in that study high-risk individuals were not selected, but subjects from a random population-based cohort were used and the underlying mechanisms were not examined either and the conclusion that IgM to oxLDL has a protective role was regarded as speculative^32^.

FOB cells are shown to promote atherosclerosis via antigen presentation to T_FH_ cells and this interaction further promotes humoral responses by stimulating B cells^50^. In contrast, MZB cells limit an exaggerated adaptive immune response via T_FH_ cell regulation^51^. In spite of the fact that CT-1 deficiency in accelerated atherosclerosis induced FOB cell production while at the same time promoting MZB cell reduction in the SP, Apoe^−/−^ct-1^−/−^ mice on HCD did not exhibit an increase in T_FH_ cells, but showed a pronounced increase in Tregs and Bregs, and reduced atherosclerotic lesion size. Taken together, these findings indicate that inhibition of CT-1 expression in atherosclerosis promotes direct anti-inflammatory effects associated with an increase of atheroprotective immune cell populations.

Chronic inflammation driven by immune cells and cytokines plays a crucial role in the development and progression of atherosclerosis. CT-1 could enhance atherogenesis via induction of inflammatory and proatherogenic molecule expression^8,26–28^. Consistently with these findings, inhibition of CT-1 expression in Apoe^−/−^ mice in accelerated atherosclerosis resulted in inducing substantial changes in the systemic levels of several cytokines and chemokines. Like many other cytokines, IL-3 and IL-6 have both proinflammatory and anti-inflammatory properties. CT-1 deficiency in Apoe^−/−^ mice on HCD resulted in elevated IL-3 systemic levels. IL-3 is secreted by activated T cells and stimulates multiple hematopoietic cell types involved in the immune response^52^, while IL-3 treatment in mice prevents mAb/LPS-induced arthritis by inhibiting inflammation^53^. Although IL-6 is a marker for systemic activation of proinflammatory cytokines^54^, it can act in an atheroprotective manner by increasing cholesterol efflux to apolipoprotein A1 (apoA1) in macrophages^55^. Importantly, Apoe^−/−^ct-1^−/−^ mice on HCD exhibited about a 2-fold increase in circulating levels of IL-6, but also a prominent reduction of cholesterol levels. Although IL-9, which is prominently elevated in Apoe^−/−^ct-1^−/−^ mice fed HCD, could be linked to the increase in Tregs in the SP of Apoe^−/−^ct-1^−/−^ mice fed HCD, as IL-9 is shown to protect Tregs against apoptosis and enhance their function^56^. Furthermore, abrogation of CT-1 in Apoe^−/−^ mice on HCD upregulated the systemic levels of IL-15. Van Es *et al*. demonstrated that while IL-15 vaccination reduced plaque size, it was accompanied by an increase in macrophages, making the plaques more vulnerable and unstable^57^. Interestingly, IL-27 is elevated in Apoe^−/−^ct-1^−/−^ mice fed HCD, and it could be linked with the atheroprotective role that IL-27 plays by regulating macrophage activation^58^. CT-1 deficiency in accelerated atherosclerosis promotes several cytokines with atheroprotective activities, like inflammation inhibition, plaque stabilization and control of macrophage activation, the net effect of which could partly explain anti-inflammatory effect observed after CT-1 abrogation in Apoe^−/−^ct-1^−/−^ mice fed HCD.

Chemokines not only direct leukocytes to sites of inflammation during atherogenesis, they also play a role in cell homeostasis and foam cell formation. For example, CXCL5, which was remarkably elevated in Apoe^−/−^ct-1^−/−^ mice fed HCD, has been shown to modulate macrophage activation, increase expression of the cholesterol efflux regulatory protein ABCA1, and enhance cholesterol efflux activity in macrophages^59^. Therefore, the increase in CXCL5 levels in Apoe^−/−^ct-1^−/−^ mice could be considered as an additional mechanism contributing to the reduction of total cholesterol as well as LDL-C. MCP-3 was shown to promote VSMCs proliferation^60^ and increase plasma total cholesterol, atherosclerotic lesions, and hepatic lipid accumulation under atherogenic conditions^61^. In agreement with these findings, we observed an increase in MCP-3 levels in the circulation upon feeding Apoe^−/−^ mice HCD, however, MCP-3 was significantly reduced in Apoe^−/−^ct-1^−/−^ mice fed HCD. MI1α and MI1β levels were elevated in Apoe^−/−^ct-1^−/−^ mice in accelerated atherosclerosis. MI1α (CCL3) deficiency in Ldlr^−/−^ mice had no effect on atherosclerotic lesion formation in the aortic sinus^62^. The results demonstrate that CT-1 deficiency in accelerated atherosclerosis promotes the induction of anti-inflammatory mediators. Taken together, these results could partly explain the beneficial effect of CT-1 deficiency in atherosclerosis progression and development. CT-1 expression in the heart and liver, in immune and endothelial cells, and in VSMCs in the aorta of ageing Apoe^−/−^ mice further highlights the involvement of CT-1 in the pathogenesis of atherosclerosis.

The present study undoubtedly demonstrates that CT-1 abrogation in accelerated atherosclerosis prevents atherosclerosis progression and development. A crucial question remains whether the presented atheroprotective effects of CT-1 deficiency in mice might be achieved in patients. Armed with a better understanding of the multiple atheroprotective effects mediated by CT-1 abrogation in accelerated atherosclerosis, we believe that CT-1 could be an efficient therapeutic target in patients with coronary atherosclerosis.

## Methods

### Mice

Eleven-week old male Apoe^−/−^ C57Bl/6 and Apoe^−/−^ct-1^−/−^ mice (crossed for 5 generations) were fed a normal chow diet (NCD, spontaneous atherosclerosis) for 16 weeks or a high-cholesterol diet (HCD) for 11 weeks (20.1% fat, 1.25% cholesterol, Research Diets, Inc., New Brunswick, NJ)^63,64^. Whole blood was collected and allowed to clot, after centrifugation of the liquid component, the serum was transferred into clean tubes and stored at −80 °C until used. Serum triglycerides, total cholesterol, low-density lipoprotein (LDL) and high-density lipoprotein (HDL), free fatty acids were measured with a high-performance liquid chromatography (HPLC) method. The lipid evaluation was performed using: TC (total cholesterol), TG (triglyceride), HDL-C (high-density lipoprotein-cholesterol), and LDL-C (low-density lipoprotein-cholesterol), calculated with the Friedewald formula taking into account that the TG levels were less than 4.5 mmol/L (Friedewald formula, in mmol/L: LDL-C 1⁄4 TC - HDL-C - TG/2.2)^65^. Experimental protocols and procedures were reviewed and approved by the Institutional Animal Care and Use Committee of the Geneva University School of Medicine (protocol number: GE77/16). Animal care and experimental procedures were carried out in accordance with the guidelines of the Institutional Animal Care and Use Committee of the Geneva University School of Medicine.

### Flow cytometry

For multi-color flow cytometry analysis, single-cell suspension (1 × 10^6^ cells) of the spleen (SP) or peripheral lymph nodes (PLN) was incubated with anti-mouse FcRIIB/FcRIIIA mAb (BD Bioscience) to avoid nonspecific staining, and subsequently stained with fluorophore-conjugated antibodies (CD4 AF488, clone RM 4–5; CD25 BV605, clone M-A251; B220 AF488, clone RA-3-6B2; IgM PE/CF594, clone R6-60.2; CD43 APC, clone S7; CD5 PerCP, clone 53–7.3; CD1d PE, clone 1B1, BD Biosciences, GATA3 BV421, clone L50-823; IL-4 AF488, clone 11B11, ROR-γ PE, clone Q13-378; IL-17 APC, clone PAJ-17R; T-bet BV605, clone 4B10; IFN-g PEcy7, clone XMG1.2) at 4 °C using predetermined optimal concentrations. Dead cells were excluded based on forward and side scatter profiles. Lymphocytes were stimulated *in vitro* with PMA (50 ng/ml; Sigma-Aldrich) and ionomycin (Sigma-Aldrich), in the presence of brefeldin A (Sigma-Aldrich) for 4 hrs and fixed and permeabilized using the Cytofix/Cytoperm Plus Fixation/Permeabilization Kit (BD Biosciences). Permeabilized cells were stained with antibodies against intracellular targets of interest (FoxP3 PE CF594, clone MF23, BD Biosciences; TGF-β PerCPCy5.5, TW7-16B4, Biolegend). After intracardial perfusion, the aorta was surgically excised and digested for 1 hour at 37 °C in with Collagenase P, dispase and DnaseI. The cell suspension was passed through a 40 μm cell strained and stained with LIVE/DEAD Fixable Near-IR Dead Cell Dye, Hoechst and anti-mouse CD4 Alexa Fluor 700, Clone RM4-5, CD31 APC, Clone MEC 13.3 BD Biosciences and CD68 PerCP/Cyanine 5.5, clone FA-11, Biolegend. After fixation and permeabilization using the Cytofix/Cytoperm Plus Fixation/Permeabilization Kit and the aorta cell suspension was stained with antibodies against intracellular targets alpha-Smooth Muscle Actin Alexa Fluor 488, clone 1A4, Thermofisher and mouse CT-1 antibody AF438, R&D and secondary antibody donkey anti-goat PE, Abcam. Samples were acquired in Gallios™ flow cytometer (Beckman Coulter) and analyzed using FlowJo software (TreeStar, Version 10.0.8r1, USA).

### Immunohistochemistry

Mouse aortic sinus was serially cut in 5 μm transversal sections, as previously described^66,67^. Sections from mouse specimens were fixed in acetone and immunostained with specific anti-mouse MMP-9 antibody (R&D Systems), anti-mouse α-SMA antibody (Thermo Fischer), anti-mouse Ly6G (BD Pharmingen) and anti-mouse CD68 (Serotec) staining in atherosclerotic roots. Quantification was performed using the MetaMorph or Definiens software. Results for other parameters were calculated as percentages of the stained area on total lesion area.

### Necrotic core and Fibrous cap thickness evaluation

To evaluation the necrotic core (NC), the criteria used by Thim *et al*.^68^ were applied. NC was considered the areas within a lesion that were negative for Picrosirius red staining (i.e., extracellular matrix was absent in parallel with total or almost complete loss of collagen). Boundary lines were delineated around those regions and the area was measured by Fiji ImageJ image analysis softwareMeasurements were performed blindly to the study groups. NC percentage area was calculated by dividing total NC area by total lesion area. Fibrous cap thickness was assessed from the largest necrotic cores stained with Picrosirius red staining and measuring the thinnest section of the cap as determined by the distance between the outer edge of the cap and the NC border. Fibrous caps buried in deep necrosis areas were not considered. Measurements were performed blindly to the study groups.

### Oil Red O staining for lipid content

Five sections per mouse aortic roots and abdominal aorta were stained with Oil Red O, as previously described^66,67^. Sections and aortas were counter-stained with Mayer’s hemalum solution and rinsed in distilled water. Quantification was performed using the MetaMorph software. Data were calculated as the percentage of the stained area from total lesion area.

### Picrosirius red staining

Five sections per mouse aortic sinus were rinsed with water and incubated with 0.1% Sirius red (Sigma Chemical Co, St Louis, MO, USA) in saturated picric acid for 90 min. Sections were rinsed twice with 0.01 M HCl for 1 min and then immersed in water. After dehydration with ethanol for 30 seconds and cover-slipping, pictures of the sections were taken with ordinary polychromatic microscopy with identical exposure settings. Total collagen content was evaluated under polychromatic light^69,70^. Quantifications were performed with Definiens Developer 2.7 software (Definiens Inc). Data were calculated and presented as the percentage of the stained area on total lesion area.

### Multiplex immunoassay

Serum samples were centrifuged, and ProcartaPlex Mouse Cytokine/Chemokine Plane 1A plex (Thermo Fisher Scientific Cat. No. EPX360-26092-901) and ProcartaPlex Mouse Antibody Isotyping Panel (Thermo Fisher Scientific Cat. No. EPX070-20815-901) kits were used. The assay procedure was performed according to the manual instructions of ProcartaPlex Multiplex Immunoassay for Simplex Kits and Combinable Panels and Mouse Isotyping Assay, respectively. Results were obtained by calibrated Luminex Instrument (Luminex Corporation). Absolute quantification was performed with xPONENT® 4.2 for MAGPIX® (Luminex Corporation).

### Measurement of ox-LDL–specific IgM Abs

The ox-LDL–specific Abs were measured using ELISA. Briefly, copper-oxidized human LDL and native human LDL (Thermo Fisher) were used to coat 96-well ELISA plates at 50 μl of 10 μg/ml overnight at 4 °C. Duplicate samples of 50 μl mouse serum diluted 1:80 were added into the ELISA plates for 1 h at 37 °C after blocking with 2% BSA, 5% FBS, followed by addition of anti-mouse IgM Abs conjugated with HRP (BD Pharmingen). Color development was done by addition of TMB solution, and plates were read at 450 nm wavelength. ox-LDL–specific Ab was determined by subtracting the native LDL OD from the ox-LDL OD.

### Protein extraction and western blot analysis

Liver, heart and SP of WT, ct-1^−/−^, Apoe^−/−^ and Apoe^−/−^ct-1^−/−^ mice were dissected, cut into pieces and lysed with lysis buffer containing CHAPS solution (Sigma) and complete mini inhibitors. Denaturized proteins were electrophoresed on NUPAGE 4–12% BT GEL 1.5 mm (Thermos Fisher), followed by transfer onto nitrocellulose membranes using a semi-dry transfer method (iBlot™ 2 Transfer Stacks, Thermo Fisher) followed by blocking. Blots were incubated with primary antibodies against CT-1 antibody AF438 (1:1000; R&D) or β-Tubulin. The bound antibodies were detected by the respective horseradish peroxidase-conjugated secondary antibodies and visualized with SuperSignal™ West Pico PLUS Chemiluminescent Substrate kit (Thermo Scientific). The resulted chemiluminescence was measured by using iBright Western Blot Imaging Systems (Thermo Scientific).

### Statistical analysis

Statistics were performed using GraphPad Prism 8. For comparison of two groups of continuous variables, two-tailed unpaired Mann-Whitney *U*-tests with a confidence level of 95% were conducted if data were non-normally distributed. For multiple group comparison, two-tailed two-ways ANOVA was performed. The number of mice used for each analysis is indicated in the figure legends. All data are presented as the mean ± SEM and the statistical significance threshold used is p ≤ 0.05. *p ≤ 0.05; **p ≤ 0.005; ***p ≤ 0.0005.

## Supplementary information

Supplementary Figures.
