# Supplementary material for: Cardiotrophin-1 Deficiency Abrogates Atherosclerosis Progression
Source: Sci Rep. 2020 Apr 1;10:5791. doi: 10.1038/s41598-020-62596-6 (PMC7113288; doi:10.1038/s41598-020-62596-6)
Supplement: Supplementary file 1 — Supplementary Figures. [file 41598_2020_62596_MOESM1_ESM.pdf]

Supplemental Material

Cardiotrophin-1 Deficiency Abrogates Atherosclerosis Progression

Kapka Miteva[1], Daniela Baptista[1], Fabrizio Montecucco[2,3], Mohamed Asrih[1], Fabienne Burger[1], Aline Roth[1], Rodrigo A. Fraga-Silva[4], Nikolaos Stergiopulos[4], François Mach[1], Karim J. Brandt[1]*

[1]Division of Cardiology, Foundation for Medical Research, Department of Medicine Specialized Medicine, Faculty of Medicine, University of Geneva, Av. de la Roseraie 64, CH-1211 Geneva 4, Switzerland. [2]Ospedale Policlinico San Martino Genoa – Italian Cardiovascular Network, 10 Largo Benzi, Genoa 16132, Italy. [3]First Clinic of Internal Medicine, Department of Internal Medicine and Centre of Excellence for Biomedical Research (CEBR), University of Genoa, 6 viale Benedetto XV, Genoa 16132, Italy. [4]Institute of Bioengineering, Ecole Polytechnique Fédérale de Lausanne, Lausanne, Switzerland.

Correspondence to Dr. Karim Brandt, Division of Cardiology, Department of Internal Medicine, Faculty of Medicine, University of Geneva, Av de la Roseraie 64, CH-1211 Geneva 14, Switzerland. Phone: +41 22 37 9 46 47, E-mail: karim.brandt@hcuge.ch

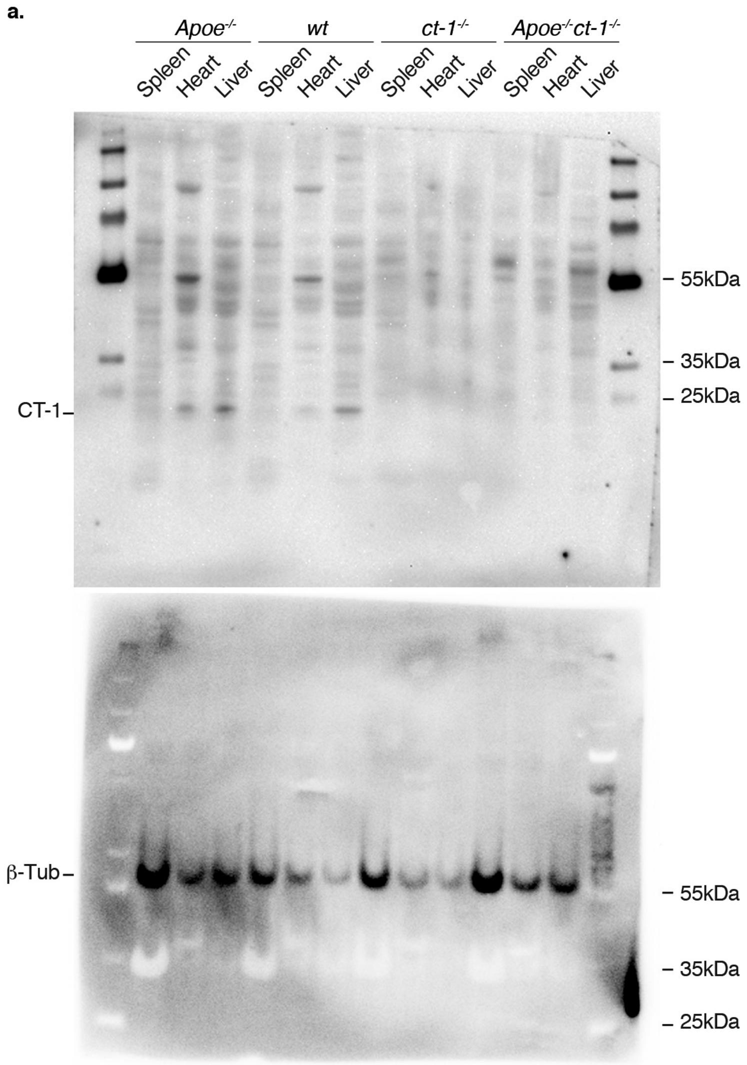

Fig. S1: Western blot analysis for CT-1 and β-tubulin in different tissues as indicated.

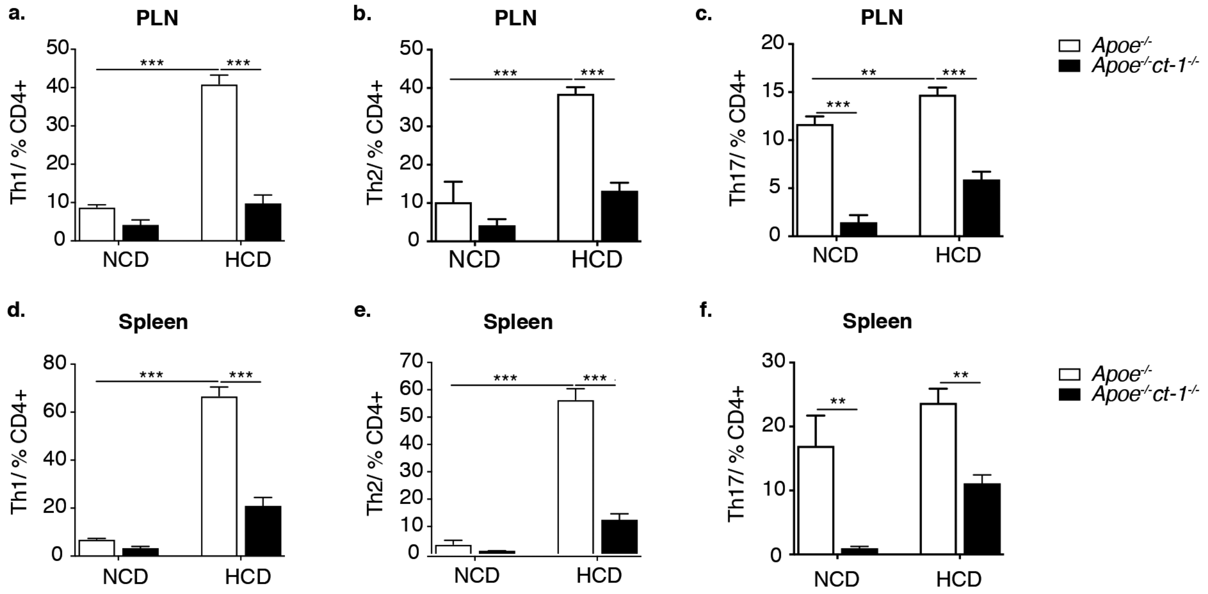

Fig. S2: CT-1 Deficiency Affect T cells subsets. Bar graphs represent the mean ± SEM of flow cytometry analysis of (**A**) Th1 percentages; (**B**) Th2 and (**C**) Th17 in the PLN and (**D**) Th1; (**E**) Th2 percentage and (**F**) Th17 in the spleen- of Apoe-/- or Apoe-/-ct-1-/- mice on NCD or HCD, as indicated, with n = 6-8/group, $^{**}p < 0.01$ and $^{***}p < 0.001$.

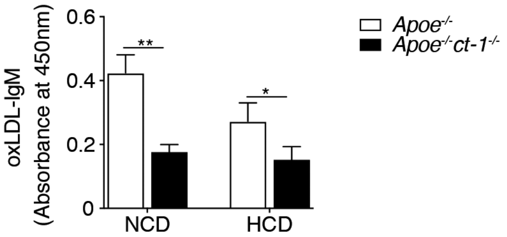

Fig. S3: Bar graphs represent the mean ± SEM of oxLDL-IgM in the serum of Apoe-/- and Apoe-/-ct-1-/- mice on NCD or HCD, with n = 6-8/group, *p < 0.01 and **p < 0.01.
